# The Incidence, Severity and Risk Factors of Renal Injury in Lung Cancer Patients Receiving Osimertinib Therapy: A Real‐World Study

**DOI:** 10.1002/cam4.71382

**Published:** 2025-11-21

**Authors:** Jieshan Lin, Xiaoying Dong, Wenfang Tang, Shuangxin Liu

**Affiliations:** ^1^ Department of Nephrology, Guangdong Provincial People's Hospital (Guangdong Academy of Medical Sciences) Southern Medical University Guangzhou Guangdong P. R. China; ^2^ Department of Nephrology, Blood Purification Center Zhongshan City People's Hospital Zhongshan Guangdong P. R. China; ^3^ School of Medicine, South China University of Technology Guangzhou Guangdong P. R. China; ^4^ Department of Cardiothoracic Surgery Zhongshan City People's Hospital Zhongshan Guangdong P. R. China; ^5^ Guangdong Lung Cancer Institute, Guangdong Provincial People's Hospital (Guangdong Academy of Medical Sciences) Southern Medical University Guangzhou Guangdong P. R. China

**Keywords:** lung cancer, osimertinib, renal injury

## Abstract

**Aim:**

The high burden of lung cancer in developing countries has led to the widespread use of EGFR inhibitors like osimertinib. However, little is known about the prevalence and risk factors of renal injury associated with osimertinib use. Therefore, this study aims to investigate the incidence, severity and risk factors of renal injury in patients receiving osimertinib in the real world.

**Methods:**

We retrospectively analyzed 1138 patients with lung cancer treated with osimertinib. Renal injury was defined as an estimated glomerular filtration rate (eGFR) below 60 mL/min/1.73m^2^ and/or the presence of proteinuria. Multivariable logistic regression models were used to identify independent risk factors for renal injury.

**Results:**

Totally, 215 (18.89%) patients developed renal injury during follow‐up. Most cases of renal injury were transient (149/1138, 13.09%), while sustained renal injury accounted for 5.8% (66/1138) of the total cohort. Impaired renal function (eGFR < 60 mL/min/1.73m^2^) was observed in 136 (11.95%) patients, predominantly at a moderate stage (eGFR 30–59), and proteinuria was present in 100 (8.79%) patients, primarily of mild severity. Among patients with renal injury, 73.53% had a mild creatinine elevation (< 50% increase from baseline). Moreover, the mean time to renal injury onset was 7.92 months (SD 8.34), with a mean recovery time of 4.21 months (SD 6.31). Importantly, age ≥ 60 years (OR = 2.307, 95% CI: 1.348–3.947, *p* = 0.002) and baseline renal injury (OR = 20.942, 95% CI: 8.398–52.223, *p* < 0.001) were significant independent risk factors for sustained renal injury.

**Conclusions:**

This study demonstrates that renal injury is not rare in lung cancer patients treated with osimertinib, particularly in patients aged ≥ 60 years or with pre‐existing renal injury. Although most cases are reversible, regular monitoring of renal function is strongly recommended for these patients.

## 1 Introduction

Lung cancer is the malignant tumor with the highest global incidence and mortality, of which non‐small cell lung cancer (NSCLC) constitutes approximately 85% of cases [1, 2]. The incidence of EGFR mutation (most commonly found in exons 18–21) in patients with NSCLC is approximately 51% [3]. Tumors with EGFR mutations are highly sensitive to EGFR tyrosine kinase inhibitors (EGFR TKIs), including afatinib, erlotinib, gefitinib, and osimertinib. As a third‐generation EGFR TKI, osimertinib demonstrates breakthrough survival benefits and has become the standard first‐line treatment for NSCLC patients with common EGFR mutations [4].

Following treatment with osimertinib, the most common treatment‐related adverse events include diarrhea, rash, nausea, and decreased appetite [5]. Studies have found that the osimertinib metabolites are excreted via both hepatic and renal routes (approximately 68% in feces and 14% in urine). Consequently, toxicity associated with osimertinib occurred more frequently in patients with impaired renal function [6]. An increasing number of cases of renal toxicity after treatment with osimertinib have recently been reported. Li et al. [7] reported that an elderly female patient developed acute renal insufficiency, hyperuricemia, metabolic acidosis and electrolyte disturbances after 5‐month treatment with osimertinib and bevacizumab. The patient was diagnosed with osimertinib‐associated rhabdomyolysis, and her symptoms improved after discontinuing osimertinib. Another report described two fatal cases of acute renal failure following treatment with a combination of osimertinib (80 mg daily) and savolitinib (600 mg daily) [8]. Additionally, Niitsu et al. [9] reported a case of biopsy‐proven renal injury, featuring mild IgA deposition, crescent formation, and tubular injury, which was attributed to osimertinib. The patient's renal function partially recovered after dose reduction from 80 to 40 mg/day. These cases illustrate that osimertinib‐related renal injury may exhibit characteristics of dose dependence and partial reversibility. Therefore, early identification and dynamic monitoring are crucial.

However, little is known about the incidence of osimertinib‐associated renal injury or its associated risk factors. Therefore, the aim of this study was to investigate the incidence, severity and risk factors of renal injury in patients receiving osimertinib.

## 2 Materials and Methods

### 2.1 Patients

This retrospective observational cohort study involved lung cancer patients treated with osimertinib at Guangdong Provincial People's Hospital between January 2017 and December 2024.

**FIGURE 1:**
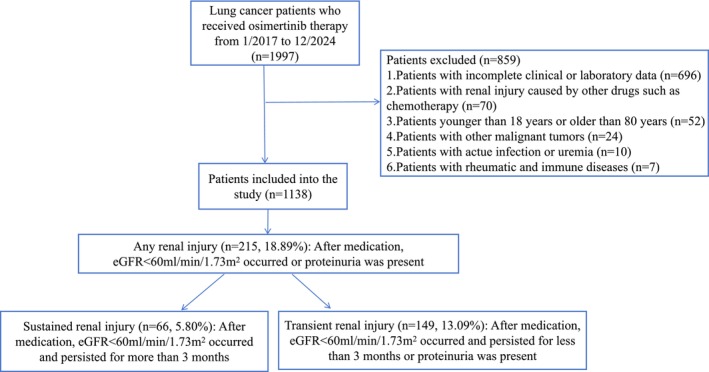
The flow chart shows how patients were selected for the present study.

The flowchart of the study design is shown in Figure 1. Inclusion criteria were: (1) age 18–80 years; (2) histological confirmation of NSCLC with an EGFR mutation; (3) treatment with osimertinib; (4) availability of baseline and follow‐up renal function data. Exclusion criteria were: (1) age < 18 years; (2) incomplete clinical or laboratory data; (3) renal injury attributable to other causes (e.g., other nephrotoxic drugs or chemotherapy); (4) history of other malignant tumors; (5) active infections, acute kidney injury before therapy, rheumatic and immune diseases; (6) dialysis prior to osimertinib initiation. In our patient population, although osimertinib was utilized in both first‐line and later‐line settings, it was consistently administered as monotherapy. The study involving human participants was approved by the Ethical Committee of Guangdong Provincial People's Hospital (KY2024‐1107‐01). The need for obtaining written informed consent from participants was waived by the aforementioned ethics committee, as the research involved no more than minimal risk and utilized pre‐existing, anonymized data.

### 2.2 The Definitions of Renal Injury

The glomerular filtration rate (eGFR) was calculated using the CKD‐EPI (Chronic Kidney Disease Epidemiology Collaboration) equation. Baseline serum creatinine (SCr) and eGFR were measured within the 3 months before osimertinib initiation. Renal injury refers to patients with eGFR less than 60 mL/min/1.73m^2^ and/or the presence of proteinuria. Patients were classified into the non‐renal injury group if they met both of the following criteria: eGFR ≥ 60 mL/min/1.73m^2^ and no evidence of proteinuria. Sustained renal injury was defined as an eGFR below 60 mL/min/1.73 m^2^ persisting for more than 3 months. All other instances of renal injury (including those with proteinuria alone or a shorter duration of eGFR decline) were categorized as transient [10].

### 2.3 Data Collection

For all patients, we collected clinical data, including age, gender, body mass index (BMI), baseline SCr and eGFR, blood pressure, EGFR mutation types, and concomitant diseases (such as hypertension, diabetes, coronary heart disease, cerebrovascular disease, and kidney disease).

### 2.4 Statistical Analysis

All analyses were performed using SPSS (version 25.0; SPSS Inc., Chicago, IL, USA). The data were presented as counts and percentages for categorical variables, median with interquartile range (IQR) for non‐normally distributed data and mean ± standard deviation (SD) for normally distributed continuous variables. Categorical variables were compared using the Chi‐square test or Fisher exact test. Continuous variables were analyzed by the Wilcoxon rank‐sum test. Univariate and multivariate logistic regression analyses were used to determine the risk factors of renal injury. Variables with a *p* value < 0.05 in the univariate logistic regression analysis were included in the multivariate logistic regression analysis. All comparisons are two‐tailed, with *p* < 0.05 considered significant.

## 3 Results

### 3.1 Demographics and Clinical Characteristics

This study initially identified 1997 lung cancer patients treated with osimertinib from January 2017 to December 2024 (Figure 1). After excluding 859 patients who did not meet the inclusion criteria, a total of 1138 patients were included in the final analysis. The cohort had a mean age of 59.24 years (SD 10.42) and 38.31% were male. The baseline serum creatinine level was 75.41 ± 20.18 μmol/L, and 26 (2.29%) patients had a baseline eGFR of < 60 mL/min/1.73m^2^. All patients were treated with osimertinib as a single agent, which was administered as first‐line treatment in 62.74% of cases; the remaining 37.26% had received prior anti‐tumor therapies, such as chemotherapy or immune checkpoint inhibitors. The baseline characteristics of the study population are summarized in Table 1. The clinical characteristics and laboratory parameters of the study population after the exclusion of patients with hypertension and diabetes can be found in Table S1.

**TABLE 1:** Clinical characteristics and laboratory parameters of the study population.

Characteristics	All patients	Non‐renal injury	Any renal injury	*p*	Transient renal injury	Sustained renal injury	*p*
Number of cases	1138	923	215		149	66	
Age, years	59.24 ± 10.42	58.21 ± 10.23	63.68 ± 10.10	**< 0.001**	62.21 ± 10.63	66.99 ± 7.87	**0.001**
Gender							
Male	436 (38.31%)	346 (37.49%)	90 (41.86%)	0.243	58 (38.93%)	32 (48.49%)	0.231
Female	702 (61.69%)	577 (62.51%)	125 (58.14%)		91 (61.07%)	34 (51.51%)	
Baseline creatinine (μmol/L)	75.41 ± 20.18	73.58 ± 16.21	83.23 ± 30.91	**< 0.001**	75.62 ± 19.08	100.42 ± 43.40	**< 0.001**
Baseline eGFR, ml/min/1.73 m2	88.01 ± 15.57	89.79 ± 14.29	80.36 ± 18.34	**< 0.001**	86.30 ± 15.52	66.94 ± 17.17	**< 0.001**
Hypertension	245 (21.53%)	158 (17.12%)	87 (40.47%)	**< 0.001**	56 (37.58%)	31 (46.97%)	0.229
Diabetes	77 (6.77%)	42 (4.55%)	35 (16.28%)	**< 0.001**	19 (12.75%)	16 (24.24%)	**0.045**
Coronary heart diseases	25 (2.20%)	13 (1.41%)	12 (5.58%)	**0.001**	7 (4.70%)	5 (7.58%)	0.520
Cerebrovascular diseases	14 (1.23%)	8 (0.87%)	6 (2.79%)	**0.033**	2 (1.34%)	4 (6.06%)	0.079
Kidney diseases	39 (3.43%)	6 (0.65%)	33 (15.35%)	**< 0.001**	9 (6.04%)	24 (36.36%)	**< 0.001**
Systolic pressure (mmHg)	127.99 ± 17.26	127.19 ± 16.98	130.78 ± 17.95	**0.014**	128.90 ± 17.26	135.19 ± 18.91	**0.032**
Diastolic pressure (mmHg)	79.81 ± 9.98	79.59 ± 9.99	80.59 ± 9.92	0.238	80.66 ± 9.55	80.43 ± 10.83	0.892
BMI	22.67 ± 3.11	22.58 ± 3.10	23.00 ± 3.11	0.146	22.68 ± 2.89	23.75 ± 3.50	0.550
Baseline kidney function (eGFR group)							
< 60 mL/min/1.73m^2^	26 (2.29%)	2 (0.22%)	24 (11.16%)	**< 0.001**	4 (2.68%)	20 (30.30%)	**< 0.001**
60–90 mL/min/1.73m^2^	567 (49.82%)	444 (48.10%)	123 (57.21%)		84 (56.38%)	39 (59.09%)	
≥ 90 mL/min/1.73m^2^	545 (47.89%)	477 (51.68%)	68 (31.63%)		61 (40.94%)	7 (10.61%)	
Peak creatinine during renal injury (μmol/L)	/	/	/		91.78 ± 30.61	131.95 ± 65.26	**< 0.001**
Peak BUN during renal injury (mmol/L)	/	/	/		6.18 ± 2.73	7.80 ± 3.95	**< 0.001**
BUN/creatinine ratio	/	/	/		6.15 ± 1.98	5.31 ± 1.38	**< 0.001**
Osimertinib used as first‐line	714 (62.74%)	588 (63.71%)	126 (58.60%)	0.164	88 (59.06%)	38 (57.58%)	0.838
EGFR mutations types							
EGFR 19del	558 (49.03%)	450 (48.75%)	108 (50.23%)	0.946	75 (50.33%)	33 (50.00%)	0.981
EGFR 21858R	467 (41.04%)	381 (41.28%)	86 (40.00%)		60 (40.27%)	26 (39.39%)	
Others	59 (5.18%)	49 (5.31%)	10 (4.65%)		7 (4.70%)	3 (4.55%)	
Unknown	54 (4.75%)	43 (4.66%)	11 (5.12%)		7 (4.70%)	4 (6.06%)	

*Note:* Values in bold are statistically significant (*p* < 0.05).

Abbreviations: BMI, body mass index; BUN, blood urea nitrogen; eGFR, estimated glomerular filtration rate.

### 3.2 Incidence and Characteristics of Renal Injury

**FIGURE 2:**
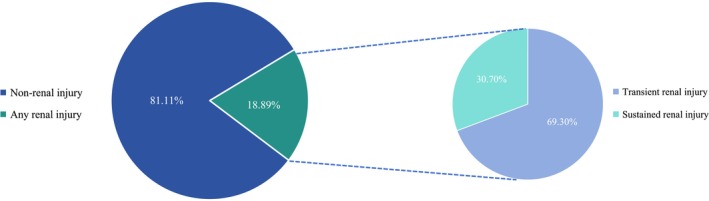
Proportions of patients with transient and sustained renal injury. The study cohort included 1138 patients, of whom 215 (18.89%) developed renal injury. Among these, 149 (69.30%) had transient injury and 66 (30.70%) had sustained injury.

During the study period, 215 (18.89%) patients developed renal injury. The proportion of patients with transient and sustained renal injury was 13.09% (149 patients) and 5.80% (66 patients), respectively (Figures 1 and 2). In a sensitivity analysis that excluded patients with hypertension and diabetes, the corresponding proportions were 10.12% and 3.37% (Figure S1).

Compared with the non‐renal injury group, patients in the renal injury group had significantly higher baseline SCr levels and a greater prevalence of hypertension, diabetes, coronary heart disease, cerebrovascular disease, and kidney disease (all *p* < 0.05). Additionally, the renal injury group had significantly lower baseline eGFR, indicating poorer kidney function (all *p* < 0.05). When comparing the sustained renal injury group to the transient group, the former comprised significantly older patients with higher baseline SCr levels, as well as a greater proportion of diabetes and pre‐existing kidney disease (all *p* < 0.05). These data are summarized in Table 1.

**FIGURE 3:**
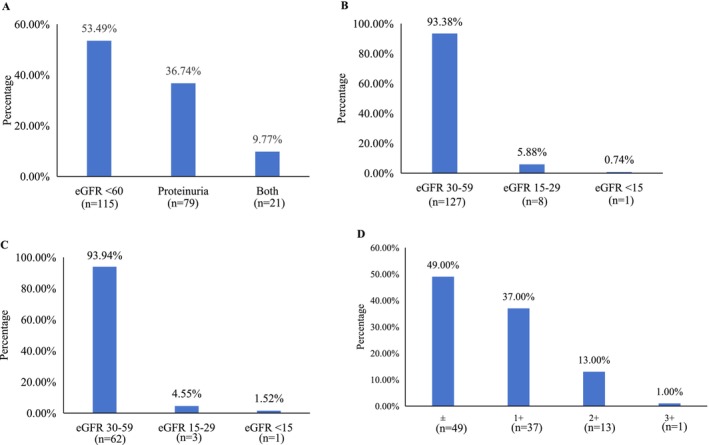
Spectrum and severity of renal injury associated with osimertinib. (A) Composition of renal injury manifestations (isolated low eGFR, isolated proteinuria, or both). (B) Distribution of eGFR stages in patients with any renal injury. (C) Distribution of eGFR stages in patients with sustained renal injury. (D) Severity grading of proteinuria in patients with any renal injury.

Figure 3 presents the types of renal injury in our study. Among the 215 affected patients, injury manifested as isolated eGFR below 60 mL/min/1.73 m^2^ (without proteinuria) in 115 (53.49%), as proteinuria with preserved eGFR (≥ 60 mL/min/1.73 m^2^) in 79 (36.74%), and as both conditions in 21 (9.77%) (Figure 3A). Of the 136 patients with elevated creatinine, eGFR was categorized as stage 3 (30–59 mL/min/1.73 m^2^) in 127 (93.38%), stage 4 (15–29 mL/min/1.73 m^2^) in 8 (5.88%), and stage 5 (< 15 mL/min/1.73 m^2^) in 1 (0.74%). A similar distribution was observed among the 66 patients with sustained injury: 62 (93.94%) in stage 3, 3 (4.55%) in stage 4, and 1 (1.52%) in stage 5 (Figure 3B,C). Among the 100 patients with proteinuria, the severity was as follows: 49 (49.0%) had trace (±) proteinuria, 37 (37.0%) had 1+, 13 (13.0%) had 2+, and 1 (1.0%) had 3+ (Figure 3D).

**FIGURE 4:**
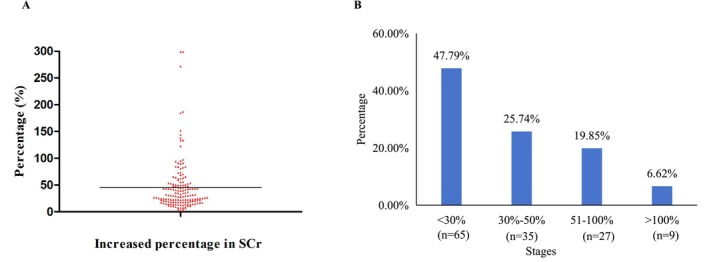
Magnitude of serum creatinine elevation in patients with osimertinib‐associated renal injury. (A) Distribution of the maximum increase in serum creatinine from baseline, categorized by percentage range. (B) Proportions of patients according to the severity of serum creatinine elevation.

The magnitude of increase in SCr among patients experiencing any renal injury is shown in Figure 4. The increase was categorized as follows: < 30% in 65 patients (47.79%), 30%–50% in 35 (25.74%), 51%–100% in 27 (19.85%), and > 100% in 9 (6.62%).

**FIGURE 5:**
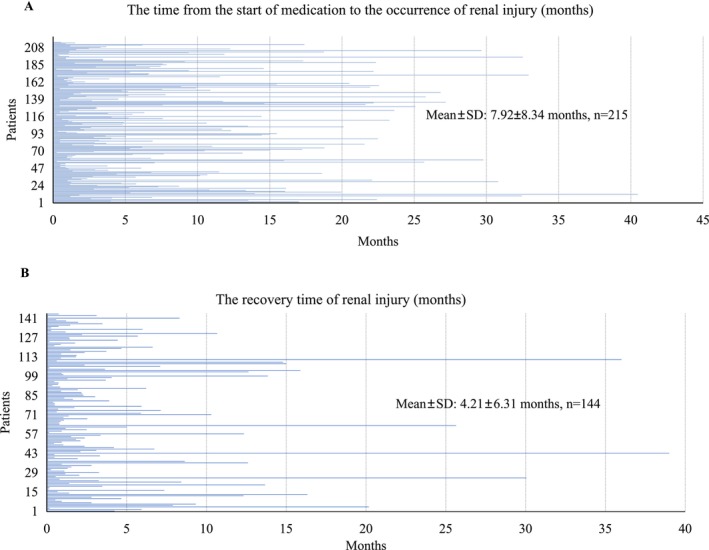
Timing of onset and recovery from osimertinib‐associated renal injury. (A) Time to onset of renal injury after initiation of osimertinib therapy. (B) Time to recovery after onset of renal injury.

The timing of onset and recovery of renal injury is presented in Figure 5. The mean time from medication initiation to the injury onset was 7.92 months (SD 8.34), and the mean recovery time was 4.21 months (SD 6.31). Recovery time was available for only 144 patients because data were either missing or because the renal injury persisted without recovery.

### 3.3 Predictors of Any Renal Injury and Sustained Renal Injury in Patients Receiving Osimertinib

The association between clinical characteristics and any renal injury in the total cohort is shown in Table 2. Univariate analysis showed that age ≥ 60 years, hypertension, diabetes, coronary heart diseases, cerebrovascular diseases and baseline renal injury (all *p* < 0.05) were significantly associated with renal injury. Multivariate logistic regression analysis identified the following as independent risk factors for renal injury: ≥ 60 years (OR = 1.737, 95% CI: 1.225–2.463, *p* = 0.002), hypertension (OR = 2.194, 95% CI: 1.517–3.173, *p* < 0.001), diabetes (OR = 2.307, 95% CI: 1.348–3.947, *p* = 0.002) and baseline renal injury (OR = 20.942, 95% CI: 8.398–52.223, *p* < 0.001).

**TABLE 2:** Logistic regression analysis between clinical characteristics and any renal injury in the total cohort.

Variables	Univariable analysis			Multivariable analysis		
	OR	95% CI	*p*	OR	95% CI	*p*
Gender						
Male	0.833	0.616–1.126	0.235			
Female						
Age group (years)						
< 60						
≥ 60	2.685	1.962–3.673	**< 0.001**	1.737	1.225–2.463	**0.002**
Osimertinib used as first‐line	1.24	0.916–1.678	0.164			
Hypertension	3.165	2.293–4.369	**< 0.001**	2.194	1.517–3.173	**< 0.001**
Diabetes	3.943	2.448–6.351	**< 0.001**	2.307	1.348–3.947	**0.002**
Coronary heart disease	4.003	1.800–8.903	**0.001**			
Cerebrovascular diseases	3.173	1.089–9.244	**0.034**			
Baseline renal fuction						
Renal injury (yes)	26.805	11.070–64.906	**< 0.001**	20.942	8.398–52.223	**< 0.001**
Renal injury (no)						

*Note:* Values in bold are statistically significant (*p* < 0.05).

The association between clinical characteristics and sustained renal injury in the cohort of patients with any renal injury is shown in Table 3. Univariate analysis showed that age ≥ 60 years, diabetes and baseline renal injury (all *p* < 0.05) were risk factors for sustained renal injury. On multivariate logistic regression analysis, age ≥ 60 years (OR = 3.363, 95% CI: 1.495–7.567, *p* = 0.003) and baseline renal injury (OR = 8.800, 95% CI: 3.655–21.186, *p* < 0.001) remained independently associated with a higher risk of sustained renal injury.

**TABLE 3:** Logistic regression analysis between clinical characteristics and sustained renal injury in the any renal injury cohort.

Variables	Univariable analysis			Multivariable analysis		
	OR	95% CI	*p*	OR	95% CI	*p*
Gender						
Male	0.677	0.378–1.215	0.191			
Female						
Age group (years)						
< 60						
≥ 60	3.775	1.786–7.979	**0.001**	3.363	1.495–7.567	**0.003**
Osimertinib used as first‐line	1.063	0.591–1.912	0.838			
Hypertension	1.514	0.840–2.729	0.167			
Diabetes	2.234	1.064–4.691	**0.034**			
Coronary heart disease	1.690	0.516–5.538	0.386			
Cerebrovascular diseases	4.820	0.860–27.008	0.074			
Baseline renal fuction						
Renal injury (yes)	8.889	3.837–20.592	**< 0.001**	8.800	3.655–21.186	**< 0.001**
Renal injury (no)						

*Note:* Values in bold are statistically significant (*p* < 0.05).

Tables S2 and S3 show that after excluding patients with hypertension and diabetes, only age ≥ 60 years and baseline renal injury remained independent risk factors for both any renal injury and sustained renal injury.

## 4 Discussion

Nowadays, lung cancer remains the leading cause of cancer‐related mortality worldwide, accounting for approximately 18.7% of all cancer deaths [2]. As a third‐generation EGFR TKI, osimertinib is the standard treatment for NSCLC patients with EGFR mutations and for those with T790M‐positive disease after prior EGFR‐TKI treatment [4]. While the most common adverse events of osimertinib are diarrhea, rash, nausea, and decreased appetite, renal injury has been reported less frequently [5]. To date, evidence regarding this issue remains limited to sporadic case reports, with a notable absence of systematic studies [7, 8, 9]. Therefore, this study aimed to systematically evaluate the incidence, severity, and risk factors of renal injury in a real‐world cohort of patients receiving osimertinib.

To our knowledge, this is the most extensive retrospective analysis examining renal injury in lung cancer patients receiving osimertinib therapy. A total of 1138 patients treated with osimertinib were finally enrolled in our study. Among the study cohort, 215 patients (18.89%) exhibited renal injury during follow‐up, with transient and sustained cases accounting for 13.09% and 5.80%, respectively.

Reduced renal function (eGFR < 60 mL/min/1.73m^2^) was observed in 136 patients (11.95%), with the vast majority exhibiting moderate impairment (eGFR 30–59 mL/min/1.73m^2^). Proteinuria was present in 100 patients (8.79%), predominantly of mild severity (< 1 g/day, trace or 1+). Among patients with renal injury, 73.53% had an increase in SCr < 50% from baseline. The mean time to renal injury onset was 7.92 months, with a mean recovery time of 4.21 months. On multivariate analysis, age ≥ 60 years, hypertension, diabetes, and baseline renal injury were identified as independent risk factors for any renal injury. In contrast, only age ≥ 60 years and baseline renal injury were independently associated with sustained renal injury.

In this study, the incidence of any, transient and sustained renal injury among patients treated with osimertinib was 18.89%, 13.09% and 5.80%, respectively. The incidence of reduced renal function (eGFR < 60 mL/min/1.73m^2^) and proteinuria was 11.95% and 8.79%, respectively. This overall incidence appears higher than the 7%–18% reported for osimertinib‐associated renal adverse events in previous studies [11, 12]. The combination therapy of osimertinib with other anti‐angiogenic drugs like bevacizumab also increased adverse events like proteinuria [13, 14]. Several factors may explain this discrepancy. First, our definition of renal injury included patients with isolated proteinuria. Second, our cohort enrolled patients with baseline renal impairment and comorbidities such as hypertension and diabetes. Third, and supporting this point, a sensitivity analysis excluding patients with hypertension and diabetes yielded lower incidences of any, transient and sustained renal injury (13.49%, 10.12% and 3.37%, respectively), underscoring the impact of these comorbidities. Fourth, 37.26% of patients had prior exposure to potentially nephrotoxic treatments like chemotherapy and immune checkpoint inhibitors. These findings suggest that the risk of renal damage from osimertinib may be underestimated in unselected real‐world populations. Therefore, regular monitoring of renal function and urinary protein is strongly recommended during osimertinib therapy.

In our study, renal injury was predominantly mild to moderate in severity, with severe renal impairment being rare (only one case each in the transient and sustained renal injury groups), which is consistent with prior reports [11, 12]. The mean time to renal injury onset was 7.75 months, with a mean recovery time of 4.21 months. Niitsu et al. [9] reported a case of osimertinib‐associated acute kidney injury (AKI) occurring 83 days after treatment initiation and response for > 6 months after osimertinib administration. Another case study described a patient receiving third‐line osimertinib‐bevacizumab combination therapy who developed AKI after 5 months of treatment, with complete symptom resolution following the withdrawal of osimertinib [7]. The kidney's vascular and tubular physiology makes it susceptible to damage from targeted agents. Based on these findings, we recommend routine monitoring of SCr and urinalysis during osimertinib treatment to facilitate early detection. Management strategies for identified injury include (1) dose reduction (e.g., to 40 mg daily), (2) temporary drug interruption until renal recovery, or (3) switching to an alternative EGFR‐TKI if nephrotoxicity persists [15]. Renal biopsy should be considered when the etiology is unclear to guide management.

Univariate and multivariate analyses identified hypertension and diabetes as independent risk factors for any renal injury, while age ≥ 60 years and baseline renal injury were risk factors for both any and sustained renal injury. After excluding patients with hypertension and diabetes, only age ≥ 60 years and baseline renal injury remained independent risk factors for both any renal injury and sustained renal injury. These results are consistent with previous research. For instance, all patients in previously reported cases of osimertinib‐associated renal injury were over 60 years of age [7, 9], and prior studies have linked baseline renal injury, advanced age and hypertension to drug‐induced nephrotoxicity [16, 17]. Furthermore, diabetes is a well‐established risk factor for the progression of AKI and CKD [18, 19]. Therefore, it is critical to increase awareness of these risk factors and implement enhanced renal function monitoring during osimertinib therapy. Clinicians should carefully assess the risk–benefit profile before prescribing osimertinib to patients with pre‐existing renal risk factors.

The specific mechanism of osimertinib‐associated renal injury is not fully elucidated. A potential pathway may involve the inhibition of EGFR, which is expressed in glomerular and tubulointerstitial compartments [20]. Supporting this, studies in animal models have shown that EGFR TKIs like erlotinib impair functional and structural recovery after renal ischemia–reperfusion injury [21], and EGFR‐mutant mice exhibit delayed renal recovery and more severe tubular injury [22]. These findings suggest that EGFR signaling is crucial for renal repair, and its inhibition may underlie osimertinib's nephrotoxic potential. Future studies are needed to investigate how osimertinib disrupts the specific molecular mechanisms of EGFR‐dependent repair in renal cells.

However, our study also had some limitations that should be considered. First, its single‐center retrospective study design is susceptible to selection bias and missing data. Second, the exclusion of patients without follow‐up creatinine values, combined with inconsistent renal function monitoring in some included patients, may have introduced heterogeneity. Third, renal biopsies were not performed; therefore, the underlying pathology of the renal injury could not be determined. A conservative management approach was chosen given the patients' advanced age, the risks associated with biopsy, and the often reversible nature of the toxicity after drug discontinuation. Finally, this study did not explore the specific mechanism of osimertinib‐associated renal injury, highlighting a need for future research to investigate the involved signaling pathways.

## 5 Conclusions

In conclusion, this study demonstrates that renal injury is a common adverse event during osimertinib therapy, though it is predominantly reversible. We identified older age (≥ 60 years), hypertension, diabetes, and baseline renal injury as key risk factors. Our results suggest that clinicians should maintain heightened vigilance in monitoring renal function among osimertinib‐treated patients, particularly those with pre‐existing risk factors, to enable real‐time adjustment.

## Author Contributions

**Jieshan Lin:** data curation (lead), investigation (equal), methodology (equal), software (lead), writing – original draft (lead), writing – review and editing (equal). **Xiaoying Dong:** data curation (equal), investigation (equal), methodology (equal), software (equal), writing – original draft (equal). **Wenfang Tang:** funding acquisition (lead), methodology (equal), resources (supporting), software (equal), supervision (lead). **Shuangxin Liu:** conceptualization (lead), funding acquisition (equal), investigation (equal), project administration (lead), resources (lead), supervision (lead), writing – review and editing (lead).

## Conflicts of Interest

The authors declare no conflicts of interest.

## Supporting information


**Figure S1:** The flow chart shows how patients were selected for the present study after excluding patients with hypertension and diabetes.

**Table S1:** Clinical characteristics and laboratory parameters of the study population after excluding patients with hypertension and diabetes.

## Data Availability

The data are available from the corresponding author on reasonable request.
