# Supplementary material for: The Incidence, Severity and Risk Factors of Renal Injury in Lung Cancer Patients Receiving Osimertinib Therapy: A Real‐World Study
Source: Cancer Med. 2025 Nov 21;14(22):e71382. doi: 10.1002/cam4.71382 (PMC12638203; doi:10.1002/cam4.71382)
Supplement: Supplementary file 1 — Figure S1: The flow chart shows how patients were selected for the present study after excluding patients with hypertension and diabetes. [file CAM4-14-e71382-s002.pdf]

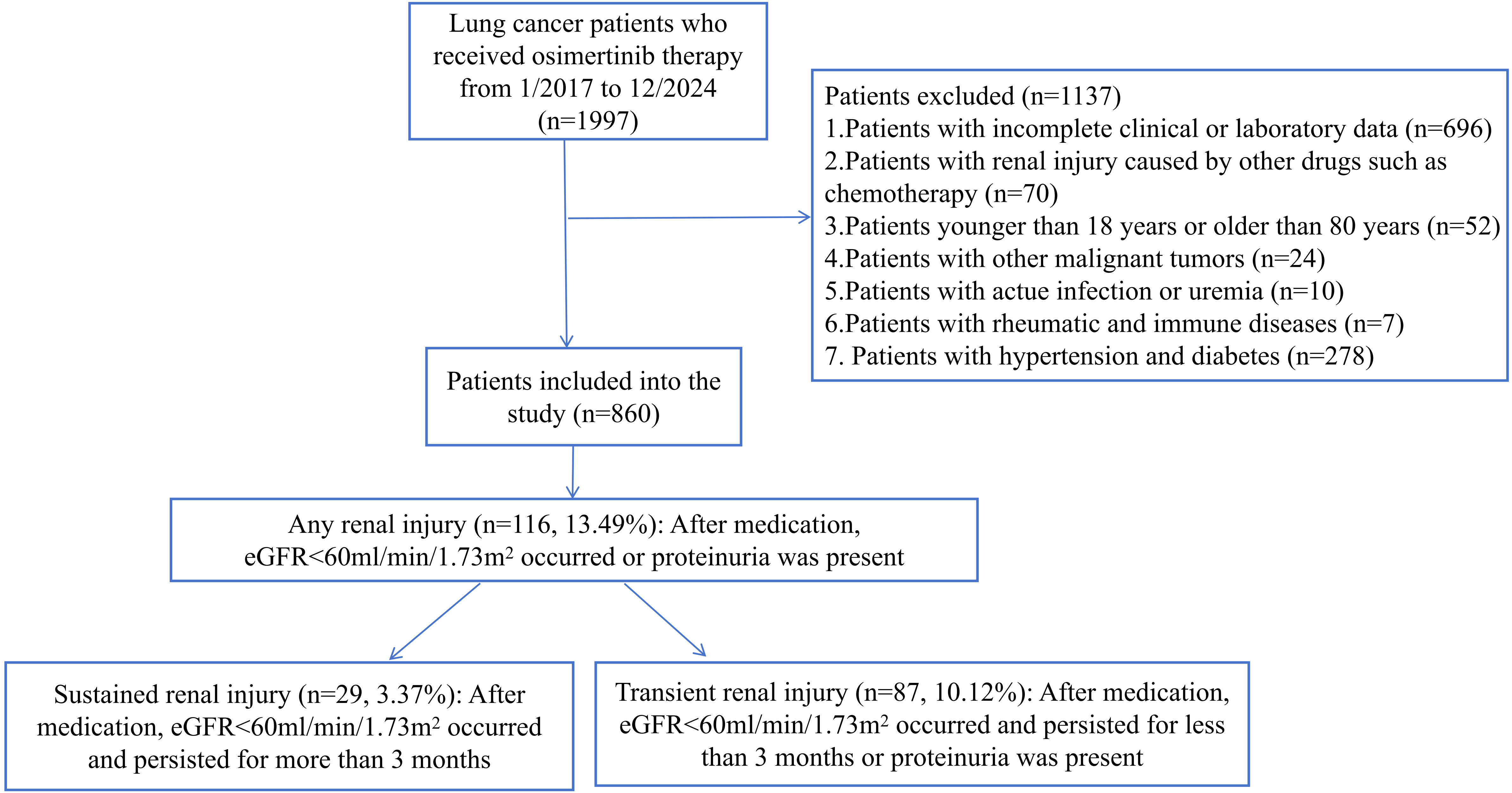

Supplementary Figure 1. The flow chart shows how patients were selected for the present study after excluding patients with hypertension and diabetes.
