# Supplementary material for: The Incidence, Severity and Risk Factors of Renal Injury in Lung Cancer Patients Receiving Osimertinib Therapy: A Real‐World Study
Source: Cancer Med. 2025 Nov 21;14(22):e71382. doi: 10.1002/cam4.71382 (PMC12638203; doi:10.1002/cam4.71382)
Supplement: Supplementary file 2 — Table S1: Clinical characteristics and laboratory parameters of the study population after excluding patients with hypertension and diabetes. [file CAM4-14-e71382-s001.docx]

| Supplementary Table 1. Clinical characteristics and laboratory parameters of the study population after excluding patients with hypertension and diabetes. | | | | | | | | | |
| --- | --- | --- | --- | --- | --- | --- | --- | --- | --- |
| Characteristics | All patients |  | Non-renal injury | Any renal injury | P value |  | Transient renal injury | Sustained renal injury | P value |
| Number of cases | 860 |  | 744 | 116 |  |  | 87 | 29 |  |
| Age, years | 57.24±10.36 |  | 56.81±10.25 | 59.99±10.68 | **0.002** |  | 58.29±10.75 | 65.09±8.82 | **0.001** |
| Gender |  |  |  |  | 0.150 |  |  |  | 0.525 |
| Male | 319 (37.09%) |  | 269 (36.16%) | 50 (43.10%) |  |  | 36 (41.38%) | 14 (48.28%) |  |
| Female | 541 (62.91%) |  | 475 (63.84%) | 66 (56.90%) |  |  | 51 (58.62%) | 15 (51.72%) |  |
| Baseline creatinine (µmol/L) | 74.52±17.36 |  | 73.38±16.24 | 81.84±22.05 | **<0.001** |  | 76.72±16.98 | 97.18±28.03 | **<0.001** |
| Baseline eGFR, ml/min/1.73m2 | 89.49±15.24 |  | 90.56±14.46 | 82.71±18.14 | **<0.001** |  | 87.56±16.01 | 68.17±16.50 | **<0.001** |
| Coronary heart diseases | 6 (0.70%) |  | 6 (0.81%) | 0 | **0.332** |  | 0 | 0 |  |
| Cerebrovascular diseases | 3 (0.35%) |  | 2 (0.27%) | 1 (0.86%) | **0.353** |  | 1 (1.15%) | 0 | 0.750 |
| Kidney diseases | 20 (2.33%) |  | 3 (0.40%) | 17 (14.66%) | **<0.001** |  | 6 (6.90%) | 11 (37.93%) | **<0.001** |
| Systolic pressure (mmHg) | 124.22±15.28 |  | 124.27±15.38 | 123.95±14.87 | 0.760 |  | 122.92±14.53 | 127.80±15.83 | 0.180 |
| Diastolic pressure (mmHg) | 78.62±9.58 |  | 78.62±9.61 | 78.59±9.47 | 0.953 |  | 78.88±8.75 | 77.50±11.99 | 0.346 |
| BMI | 22.26±2.98 |  | 22.22±3.05 | 22.48±2.61 | 0.408 |  | 22.37±2.64 | 22.81±2.55 | 0.352 |
| Baseline kidney function (eGFR group) |  |  |  |  | **<0.001** |  |  |  | **<0.001** |
| <60ml/min/1.73m^2^ | 12 (1.40%) |  | 1 (0.13%) | 11 (9.48%) |  |  | 2 (2.30%) | 9 (31.04%) |  |
| 60-90ml/min/1.73m^2^ | 410 (47.67%) |  | 347 (46.64%) | 63 (54.31%) |  |  | 46 (52.87%) | 17 (58.62%) |  |
| ≥90ml/min/1.73m^2^ | 438 (50.93%) |  | 396 (53.23%) | 42 (36.21%) |  |  | 39 (44.83%) | 3 (10.34%) |  |
| Peak creatinine during renal injury (µmol/L) | / |  | / | / |  |  | 88.17±27.75 | 125.04±40.30 | **<0.001** |
| Peak BUN during renal injury (mmol/L) | / |  | / | / |  |  | 5.69±2.53 | 7.39±2.84 | **<0.001** |
| BUN/creatinine ratio | / |  | / | / |  |  | 5.88±1.81 | 5.30±1.39 | **0.180** |
| Osimertinib used as first-line | 527 (61.28%) |  | 463 (62.23%) | 64 (55.17%) | 0.152 |  | 48 (55.17%) | 16 (55.17%) | 0.587 |
| EGFR mutations types |  |  |  |  | 0.292 |  |  |  | 0.583 |
| EGFR 19del | 417 (48.49%) |  | 357 (47.98%) | 60 (51.72%) |  |  | 47 (54.02%) | 13 (44.83%) |  |
| EGFR 21858R | 353 (41.04%) |  | 307 (41.27%) | 46 (39.66%) |  |  | 33 (37.93%) | 13 (44.83%) |  |
| Others | 47 (5.47%) |  | 39 (5.24%) | 8 (6.90%) |  |  | 5 (5.75%) | 3 (10.34%) |  |
| Unknown | 43 (5.00%) |  | 41 (5.51%) | 2 (1.72%) |  |  | 2 (2.30%) | 0 |  |
| BMI, body mass index; eGFR, estimated glomerular filtration rate; BUN, blood urea nitrogen. | | | | | | | | | |

| Supplementary Table 2. Logistic regression analysis between clinical characteristics and any renal injury in the total cohort after excluding patients with hypertension and diabetes. | | | | | | | |  |
| --- | --- | --- | --- | --- | --- | --- | --- | --- |
|  |  |  |  |  |  |  |  |  |
| Variables | Univariable analysis | | |  | Multivariable analysis | | |  |
|  | OR | 95% CI | P vale |  | OR | 95% CI | P vale |  |
| Gender |  |  |  |  |  |  |  |  |
| Male | 0.748 | 0.503-1.112 | 0.151 |  |  |  |  |  |
| Female |  |  |  |  |  |  |  |  |
| Age group (years) |  |  |  |  |  |  |  |  |
| <60 |  |  |  |  |  |  |  |  |
| ≥60 | 1.817 | 1.226-2.692 | **0.003** |  | 1.554 | 1.025-2.358 | **0.038** |  |
| Osimertinib used as first-line therapy | 1.339 | 0.902-1.987 | 0.148 |  |  |  |  |  |
| Coronary heart disease | 0.000 | 0.00 | 0.999 |  |  |  |  |  |
| Cerebrovascular diseases | 3.101 | 0.279-34.476 | 0.357 |  |  |  |  |  |
| Baseline renal function |  |  |  |  |  |  |  |  |
| Renal injury (Yes) | 40.697 | 11.715-141.374 | **<0.001** |  | 36.641 | 10.490-127.983 | **<0.001** |  |
| Renal injury (No) |  |  |  |  |  |  |  |  |

| Supplementary Table 3. Logistic regression analysis between clinical characteristics and sustained renal injury in the any renal injury cohort after excluding patients with hypertension and diabetes. | | | | | | | |  |
| --- | --- | --- | --- | --- | --- | --- | --- | --- |
|  |  |  |  |  |  |  |  |  |
| Variables | Univariable analysis | | |  | Multivariable analysis | | |  |
|  | OR | 95% CI | P vale |  | OR | 95% CI | P vale |  |
| Gender |  |  |  |  |  |  |  |  |
| Male | 0.756 | 0.326-1.759 | 0.517 |  |  |  |  |  |
| Female |  |  |  |  |  |  |  |  |
| Age group (years) |  |  |  |  |  |  |  |  |
| <60 |  |  |  |  |  |  |  |  |
| ≥60 | 5.180 | 1.917-13.996 | **0.001** |  | 5.367 | 1.833-15.712 | **0.002** |  |
| Osimertinib used as first-line therapy | 1.000 | 0.430-2.328 | 1 |  |  |  |  |  |
| Baseline renal fuction |  |  |  |  |  |  |  |  |
| Renal injury (Yes) | 8.250 | 2.697-25.239 | **<0.001** |  | 8.603 | 2.546-29.073 | **0.001** |  |
| Renal injury (No) |  |  |  |  |  |  |  |  |
